# The Predictive Role of Artificial Intelligence-Based Chest CT Quantification in Patients with COVID-19 Pneumonia

**DOI:** 10.3390/tomography7040058

**Published:** 2021-11-01

**Authors:** István Viktor Szabó, Judit Simon, Chiara Nardocci, Anna Sára Kardos, Norbert Nagy, Renad-Heyam Abdelrahman, Emese Zsarnóczay, Bence Fejér, Balázs Futácsi, Veronika Müller, Béla Merkely, Pál Maurovich-Horvat

**Affiliations:** 1 Medical Imaging Centre, Semmelweis University, 1082 Budapest, Hungary; istvan.szabo97@gmail.com (I.V.S.); juditsimon21@gmail.com (J.S.); chiara.nardocci@gmail.com (C.N.); annakardos97@gmail.com (A.S.K.); nagynorbi526@gmail.com (N.N.); heyamohd@gmail.com (R.-H.A.); emese.zsarnoczay@gmail.com (E.Z.); bence.fejer@gmail.com (B.F.); balazs.futacsi@gmail.com (B.F.); 2 MTA-SE Cardiovascular Imaging Research Group, Heart and Vascular Center, Semmelweis University, 1122 Budapest, Hungary; merkely.bela@gmail.com; 3 Department of Pulmonology, Semmelweis University, 1082 Budapest, Hungary; muller.veronika@med.semmelweis-univ.hu

**Keywords:** COVID-19, artificial intelligence, computed tomography

## Abstract

We sought to analyze the prognostic value of laboratory and clinical data, and an artificial intelligence (AI)-based algorithm for Coronavirus disease 2019 (COVID-19) severity scoring, on CT-scans of patients hospitalized with COVID-19. Moreover, we aimed to determine personalized probabilities of clinical deterioration. Data of symptomatic patients with COVID-19 who underwent chest-CT-examination at the time of hospital admission between April and November 2020 were analyzed. COVID-19 severity score was automatically quantified for each pulmonary lobe as the percentage of affected lung parenchyma with the AI-based algorithm. Clinical deterioration was defined as a composite of admission to the intensive care unit, need for invasive mechanical ventilation, use of vasopressors or in-hospital mortality. In total 326 consecutive patients were included in the analysis (mean age 66.7 ± 15.3 years, 52.1% male) of whom 85 (26.1%) experienced clinical deterioration. In the multivariable regression analysis prior myocardial infarction (OR = 2.81, 95% CI = 1.12–7.04, *p* = 0.027), immunodeficiency (OR = 2.08, 95% CI = 1.02–4.25, *p* = 0.043), C-reactive protein (OR = 1.73, 95% CI = 1.32–2.33, *p* < 0.001) and AI-based COVID-19 severity score (OR = 1.08; 95% CI = 1.02–1.15, *p* = 0.013) appeared to be independent predictors of clinical deterioration. Personalized probability values were determined. AI-based COVID-19 severity score assessed at hospital admission can provide additional information about the prognosis of COVID-19, possibly serving as a useful tool for individualized risk-stratification.

## 1. Introduction

Coronavirus disease 2019 (COVID-19), caused by severe acute respiratory syndrome coronavirus 2 (SARS-CoV-2) is associated with substantial morbidity and mortality [1]. In only one year, it has impacted over two hundred and eighteen countries with infection numbers over 60 million and deaths over 1.4 million, showing no signs of deceleration thus far [2,3]. Early risk stratification could help medical personnel in triaging infected patients and allocating limited healthcare resources. Previous studies have shown that visual scoring of the extent of lung injury depicted by computed tomography (CT) correlates well with clinical severity in patients with COVID-19 [4,5]. However, visual inspection of the CT-images might be linked with higher variability and the large number of daily CT-scans means a great challenge for the radiologists. Artificial intelligence using deep learning has been advocated for automated reading and quantification of parenchymal involvement on CT-scans, helping speed up the reading time and reducing the burden of the radiologists [6]. However, literature is heterogeneous about the predictors of mortality and the clinical deterioration in patients with COVID-19. Using a combination of AI-based CT assessment and clinical and laboratory data, the prognosis might be predicted more precisely.

Therefore, the aim of our study was to examine if baseline clinical, laboratory data and AI-based chest-CT quantification can provide prognostic information about the clinical deterioration in symptomatic patients hospitalized with COVID-19. Moreover, we aimed to determine personalized AI-based probabilities stratified by the independent predictors of COVID-19-related adverse outcomes.

## 2. Materials and Methods

### 2.1. Patient Selection and Data Collection

In our retrospective, single-center study clinical, laboratory and CT-imaging data were recorded consecutively in symptomatic patients with COVID-19 who underwent CT exam and were hospitalized after admission to the Emergency Department of our university between April and November 2020. The SARS-CoV2 positivity was determined by reverse-transcriptase polymerase chain reaction (RT-PCR) of standard nasopharyngeal and oropharyngeal swab specimens. Only symptomatic patients were included, who had at least one of the following symptoms: fever or chills, dry cough, fatigue, sputum production, shortness of breath, muscle or joint pain, sore throat, headache, gastrointestinal symptoms and loss of smell or taste. Exclusion criteria were prior pulmonectomy or lobectomy, presence of hydro- or hemothorax, or empyema with compressive atelectasis and CT-slice thickness over 2 mm.

Medical history data including age, sex, body mass index (BMI), hypertension, diabetes, dyslipidemia, prior myocardial infarction, heart failure, chronic lung disease (including asthma, chronic obstructive pulmonary disease, obstructive sleep apnea), impaired kidney function (defined as estimated glomerular filtration rate <60 mL/min/1.73 m^2^) and immunodeficiency (defined as acquired immunodeficiency resulting from various immunosuppressive agents such as chemotherapy, disease-modifying drugs and immunosuppressive drugs after organ transplants) were recorded. Blood test results including lymphocyte count, liver enzymes, lactate-dehydrogenase (LDH), C-reactive protein (CRP), ferritin, d-dimer, prothrombin time, high sensitivity troponin T, creatine-kinase and oxygen saturation (SpO2) at room air were collected at the time of hospital admission.

### 2.2. Outcome Definition

The primary outcome was a composite of admission to the intensive care unit, need for invasive mechanical ventilation or vasopressor therapy, or in-hospital death. Patients with/without primary outcome during hospitalization are referred to as patients with/without clinical deterioration.

### 2.3. CT Acquisition Protocol and Image Reconstruction

Chest CT scans were obtained using a 128-slice CT scanner (Philips Incisive, Philips Healthcare, Cleveland, OH, USA) in the supine position during inspiratory breath hold. The CT acquisition protocol included a peak tube voltage of 120 kV, automatic tube current modulation (300–500 mAs), slice thickness of 1 mm and reconstructruction increment 0.85 with a collimation of 64 × 0.625. Infection control and prevention were taken into account in all cases. Images were reconstructed using standard lung filters.

### 2.4. CT Image Analysis

**Figure 1:**
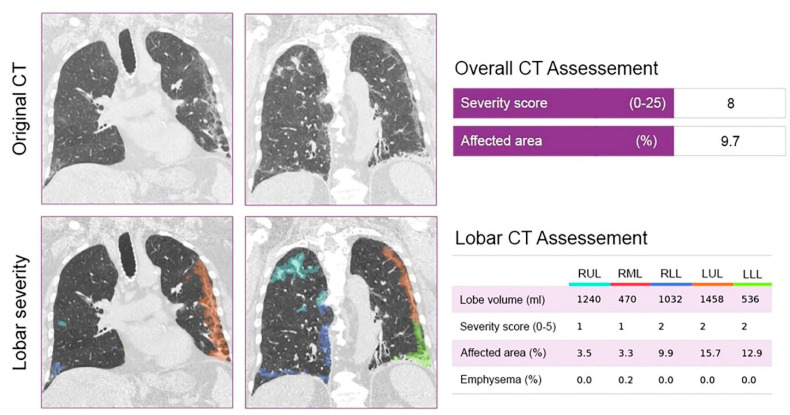
Representative example of the AI-based CAD4COVID–CT software of a patient with a total CT severity score of 8. The original and AI–assessed chest–CT of a 67–year old male patient, who was hospitalized with an SpO2 of 95% at the time of hospital admission. The patient was receiving chemotherapy for prostate cancer at the time of the CT scan. As a result of the standard therapy, the patient experienced gradual improvement in his condition during hospitalization and was released home after 10 days. CT severity scores, affected areas, lobe volumes and emphysema areas are reported on the right side. Severity scores were calculated using the percentage of the affected area of the parenchyma. Abbreviations: CT = computed tomography.

CT quantification of pulmonary parenchyma was performed using the CAD4COVID-CT software (Thirona, Nijmegen, The Netherlands). CAD4COVID-CT is an AI-based software package that is offered free-of-charge during the COVID-19 pandemic to assist healthcare professionals in their daily tasks. The software automatically quantifies the lobar extent of COVID-19 severity from inspiratory CT scans using state-of-the-art deep learning techniques. The AI software identifies the lobar regions affected by COVID-19 pneumonia and quantifies them as the percentage of total lobe volume. Each lobe will have a severity score based on the extent of affected area as following: 0 (affected area: 0%); 1 (affected area: 0.1–5.0%); 2 (affected area: 5.1–25.0%); 3 (affected area: 25.1–50.0%); 4 (affected area: 50.1–75.0%); and 5 (affected area: over 75.0%). The severity scores of each lobe are added together resulting in the total severity score. CAD4COVID-CT is CE 0344 certified as a Class IIa medical device and is permitted to be used in the US by the FDA. Representative example can be seen in Figure 1.

### 2.5. Statistical Analysis

Continuous variables were expressed as mean ± standard deviation (SD) or median with interquartile range (IQR), as deemed appropriate. Categorical variables were expressed as absolute numbers and percentages. In the descriptive statistics, continuous variables were tested with Student’s *t*-test or non-parametric Mann-Whitney U test, and categorical variables were compared with Chi-square test.

Uni- and multivariable logistic regression models were built to determine the independent associates of clinical deterioration in COVID-19. First, we applied univariable logistic regression analysis for all collected clinical parameters at the time of admission, such as age, sex, BMI, hypertension, diabetes, dyslipidemia, smoking status, prior myocardial infarction, presence of heart failure, chronic lung disease, impaired kidney function, immunodeficiency and SpO2 at room air at the time of hospital admission. Among laboratory parameters, only CRP was included in the analysis, based on previous studies [7,8]. In order to evaluate the predictive role of these parameters, two sets of models were built: Model 1 included clinical parameters that were significant in the univariable analysis and Model 2 included Model 1 + AI-based CT severity score. Based on the results of the multivariable analysis, we determined personalized probabilities for clinical deterioration, as stratified by the independent predictors. For this, we conducted simulation analysis with standard values (mean for continuous and most frequent value for categorical variables) for those variables that were not statistically significant in the final multivariable analysis, and we built several different models for each possible combination of the independent predictors of clinical deterioration. Finally, we excluded probability values of each model. Statistical analyses were performed in R environment (version 4.0.3) and two-sided *p*-value < 0.05 was considered statistically significant.

### 2.6. Ethical Approval

Ethical approval for this study was obtained from the Regional, Institutional Academic and Research Ethics Committee of our university (256/2020). Written informed consent was obtained from all subjects before the study.

## 3. Results

### 3.1. Patient Characteristics and Symptoms

**Figure A1:**
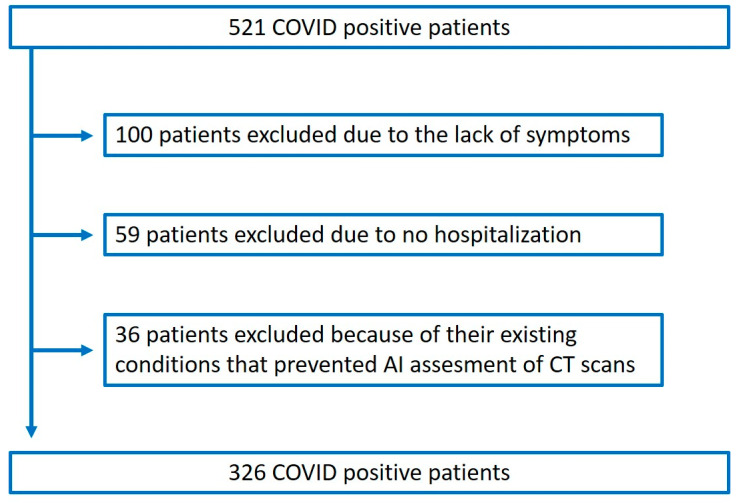
Study flowchart.

Altogether 521 patients with COVID-19 were enrolled in our study. After exclusion, 326 patients (mean age 66.7 ± 15.3 years, 52.1% male) were included in the final analyses (Figure A1 in Appendix A). Clinical deterioration was present in 85 of 326 (26.1%) patients. Anthropometric, clinical and laboratory characteristics of patients at the time of hospital admission are reported in Table 1 and Table 2. Those with clinical deterioration were older (70.0 ± 14.1 years vs. 65.5 ± 15.6 years, *p* = 0.016), had higher proportion of prior myocardial infarction (17.6% vs. 6.2%, *p* = 0.004), impaired kidney function (21.2% vs. 11.2%, *p* = 0.035) and immunodeficiency (29.4% vs. 18.3%, *p* = 0.044). Moreover, they had significantly decreased SpO2 (92% [87.0–96.0] vs. 95% [93.0–97.0]), higher LDH (448.5 U/L [286.0–627.5] vs. 241.0 U/L [192.5–339.5]), CRP (140.4 mg/L [87.6–226.7] vs. 62.8 mg/L [20.1–40.4]), ferritin (835.5 ng/L [406.8–1308.2] vs. 683 ng/L [298.0–859.0]) and d-dimer (2.50 ug/mL [1.41–4.24] vs. 1.17 ug/mL [0.62–2.62]) values at the time of hospital admission (all *p* < 0.001).

Regarding the symptoms, dry cough (51.9% vs. 35.4%, *p* = 0.011) and muscle or joint pain (15.4% vs. 6.1%, *p* = 0.036) were more prevalent in patients with a better prognosis. On the other hand, among patients with adverse outcome, shortness of breath (60.0% vs. 45.2% *p* = 0.029) was more frequent at hospital admission.

### 3.2. AI-Based CT Quantification

**Figure A2:**
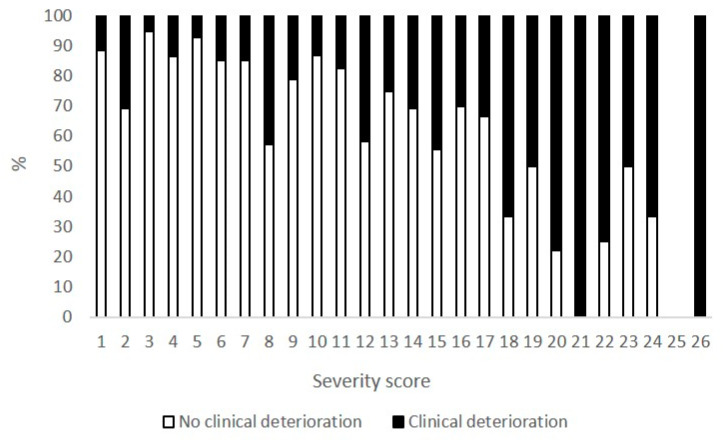
Outcome percentages stratified by different severity scores.

Patients underwent non-contrast chest CT examination at the time of hospital admission. AI-based quantitative measurements and calculated severity scores can be seen in Table 3 and Table 4 and Figure A2. Patients with future clinical deterioration had lower lung volumes in the right upper (688.0 mL [541.5–908.5] vs. 788.5 mL [628.5–942.0], *p* = 0.017), left upper (895.0 mL [725.0–1177.0] vs. 990.0 mL [796.2–1231.2], *p* = 0.029) and left lower lobes (690.5 mL [518.2–861.5] vs. 786.0 mL [589.0–2138.0], *p* = 0.016). Those with later clinical deterioration had significantly higher affected area and severity score in all five lobes at the time of hospital admission (total affected area: 21.0% [6.2–45.0%] vs. 5.6% [1.5–16.6%]; total severity score: 11.0 [7.0–17.3] vs. 6.0 [3.0–10.0], all *p* < 0.001).

### 3.3. Predictors of Adverse Outcome

Results of the uni- and multivariable logistic regression analyses are reported in Table 5. Based on prior studies, only CRP was analyzed among the laboratory parameters [7,9]. In the univariate analysis age (OR = 1.02, 95% CI = 1.00–1.04, *p* = 0.022), prior myocardial infarction (OR = 3.23, 95% CI = 1.50–7.00, *p* = 0.003), impaired kidney function (OR = 2.13, 95% CI = 1.09–4.08, *p* = 0.024), immunodeficiency (OR = 1.87, 95% CI = 1.05–3.28, *p* = 0.032), SpO2 (OR = 0.90, 95% CI = 0.86–0.94, *p* < 0.001), CRP (OR = 2.25, 95% CI = 1.76–2.96, *p* < 0.001) and AI-based severity score (OR = 1.15, 95% CI = 1.10–1.20, *p* < 0.001) were significantly associated with worse clinical outcome. Using these parameters two sets of models were built. In the clinical model prior myocardial infarction (OR = 3.31, 95% CI = 1.37–8.12, *p* = 0.008), SpO2 (OR = 0.94, 95% CI = 0.89–0.98, *p* = 0.005) and CRP (OR = 1.95, 95% CI = 1.51–2.58, *p* < 0.001) remained statistically significant. When AI-based severity score was added to the model, prior myocardial infarction (OR = 2.81, 95% CI = 1.12–7.04, *p* = 0.027), immunodeficiency (OR = 2.08, 95% CI = 1.02–4.25, *p* = 0.043), CRP (OR = 1.73, 95% CI = 1.32–2.33, *p* < 0.001) and AI-based severity score (OR = 1.08, 95% CI = 1.02–1.15, *p* = 0.013) proved to be independent predictors of clinical decline.

### 3.4. Personalized Risk Probabilities

**Figure 2:**
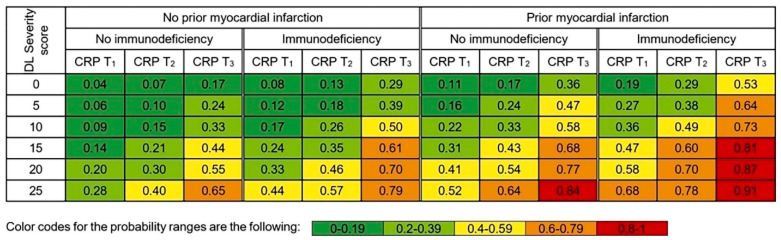
Deep learning–based probability of clinical deterioration for given severity score values as stratified by history of myocardial infarction, presence of immunodeficiency and CRP tertiles. CRP tertiles: T_1_ < 45.1 mg/L; T_2_ = 45.1–114.4 mg/L; T_3_ > 114.4 mg/L.

**Figure A3:**
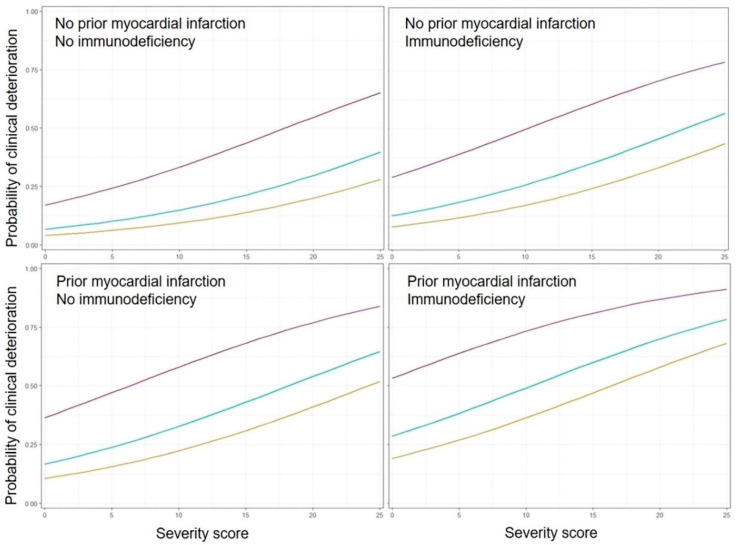
Deep learning-based probability plots for clinical decline as stratified by prior myocardial infarction, immunodeficiency, CRP and AI-based CT severity score.

We determined personalized probabilities for clinical deterioration, as stratified by the independent predictors in the multivariable analysis. Based on this, we simulated the probability of clinical deterioration for given AI-based severity score values for patients with or without prior myocardial infarction, immunodeficiency and CRP tertiles (T1 < 45.1 mg/L; T2 = 45.1–114.4 mg/L; T3 > 114.4 mg/L). Detailed results are reported in Figure 2 and probability plots can be seen in Figure A3.

### 3.5. Receiver Operating Characteristic (ROC) Curves

**Figure A4:**
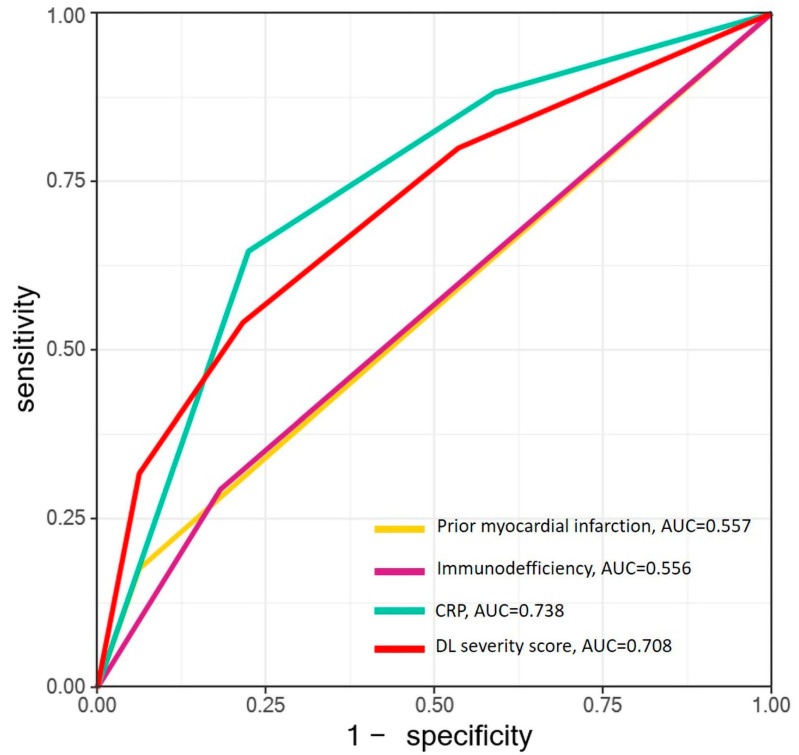
ROC analysis of independent predictors. Abbreviations: AUC = area under curve, CRP = C-reactive protein, DL = deep learning.

**Figure A5:**
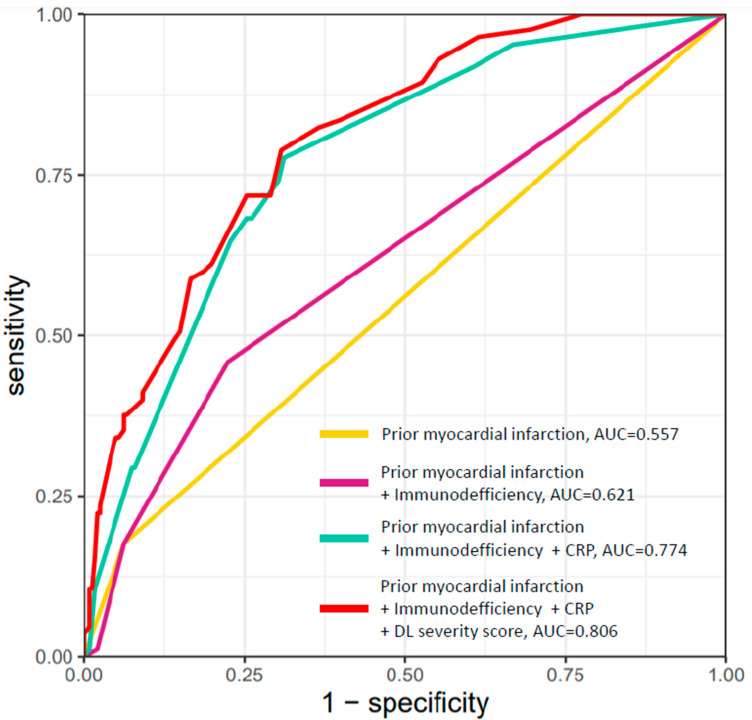
ROC analysis of combined predictors. Abbreviations: AUC = area under curve, CRP = C-reactive protein, DL = deep learning.

ROC curves were created using the following parameters: prior myocardial infarction, immunodefficiency, CRP and DL severity score, which can be seen in Figure A4 and Figure A5.

## 4. Discussion

We have demonstrated that prior myocardial infarction, immunodeficiency, CRP and AI-based severity score determined at the time of hospital admission are independent predictors of adverse clinical outcome, defined by admission to the intensive care unit, need for vasopressor or invasive mechanical ventilation and in-hospital mortality. Based on these parameters, we have determined personalized probabilities that may support clinical decision-making in triaging patients.

Early risk-stratification of patients with COVID-19 is essential, especially in large medical centers where optimal patient allocation is challenging due to limited health resources. There are no well-established predictors of clinical decline, as findings of previous studies are not consistent [10,11,12,13,14,15,16]. Our results are in line with previous studies regarding the predictive role of prior myocardial infarction, immunodeficiency and increasing CRP levels [17,18,19,20,21,22]. Previous studies reported coronary artery disease as an important early predictor for mortality in patients with COVID-19 [17,18,19,20]. Consistent with these findings, in our study population a larger proportion of patients who experienced clinical decline had myocardial infarction in their medical history. It suggests that preexisting severe coronary artery disease may aggravate myocardial injury caused by COVID-19. Moreover, systemic inflammatory status might increase inflammatory activity within the coronary artery plaques, making them more prone to rupture [23]. Therefore, comprehensive management of patients with prior myocardial infarction is important in order to improve outcome.

In our study, immunodeficiency, defined as recent cancer or immunosuppressant therapy significantly associated with worse in-hospital outcome. Previous studies stated that patients with cancer appear more vulnerable to COVID-19 [21,22]. Jee J et al. reported that even though cytotoxic chemotherapy itself was not associated with worse outcome, pre-COVID-19 neutropenia was an important risk factor for COVID-19-associated respiratory failure or death [24]. Even though prior studies did not show significant association between chemotherapy and worse outcome in patients with COVID-19, combination of chemo- and immunotherapy proved to be an independent risk factor for developing severe respiratory failure [25]. However, in our study we did not analyze neither the effect of immunosuppressant therapy or cancer itself separately, nor cancer severity on COVID-19-related outcome.

From the laboratory parameters, only CRP was built into the final multivariable analysis as it was reported among the most consistent laboratory parameters for risk prediction in prior studies [7,8]. CRP is produced by the liver as a response to inflammation [26]. Even if CRP is generally much higher in bacterial than in viral infections, patients with COVID-19 usually have markedly elevated levels [27,28]. Moreover, in our study population, more severe cases had higher CRP levels even at the time of hospital admission compared to those patients who did not experience clinical deterioration, and this association remained significant even in the multivariable analysis. These findings suggest that close monitoring of CRP levels could improve patient management and outcome.

In this study we tested an automatic AI-based CT severity score assessment. There are several advantages of AI against visual assessment by radiologists [29]. The AI-based severity score is consistent, reproducible and standardized, while prognostic scores and affected area percentages annotated by radiologists may differ vastly. The gap between the number of radiologists and the number of CT examinations is growing day by day. Based on our results, integrating the AI-based severity score into the daily practice of triaging patients with COVID-19 could greatly improve clinical outcome.

CAD4COVID can also be used on chest radiographs. In a previous study, the software was trained on 24,678 chest radiographs and 1540 scans were used for validation. The AI system classified COVID-19 pneumonia correctly with an area under the receiver operating curve of 0.81, as compared to RT-PCR test. Moreover, the system outperformed six radiologists with 5 to over 30 years of experience (*p* < 0.001) [30].

Another study also used a combination of CT and AI for differentiating COVID-19 from commonly acquired pneumonia (CAP). In a study of 4352 CT scans (29.7% with COVID-19 pneumonia), the AI had a sensitivity of 90% and a specificity of 96% for the diagnosis of COVID-19, allowing accurate detection of COVID-19 pneumonia [31]. A number of limitations of the current work need to be acknowledged. First, this is a retrospective single-center study. Second, not all patients admitted to the Emergency Department underwent chest-CT examination, and some received a chest X-ray instead. Third, the effect of treatment on the outcome was not analyzed. However, it is important to note that all patients received similar therapy based on international recommendations. Finally, the full model was not validated in external cohorts, therefore our results should be considered as hypothesis-generating and further studies are warranted to test the utility of AI-based probability estimation of clinical deterioration in COVID-19 patients.

## 5. Conclusions

In conclusion, our study demonstrated that the probability of clinical deterioration for a given AI-based severity score value increases in the presence of immunodeficiency, prior myocardial infarction and increasing CRP levels. These findings indicate that AI-based severity score of the baseline chest-CT provides additional information for the prognosis of COVID-19, apart from laboratory parameters and clinical data. Our simulation results provide personalized probabilities of adverse in-hospital outcome. These results might assist individualized decision-making in patients with COVID-19.

## Figures and Tables

**Table 1:** Clinical characteristics of patients at the time of admission.

	All Patients (*n* = 326)	No Clinical Deterioration (*n* = 241)	Clinical Deterioration (*n* = 85)	*p* Value
Age (years)	66.7 ± 15.3	65.5 ± 15.6	70.0 ± 14.1	0.016
Male, *n* (%)	170 (52.1)	126 (52.3)	44 (51.7)	1.000
BMI (kg/m^2^)	29.5 ± 6.5	29.9 ± 6.5	27.7 ± 6.0	0.126
Hypertension, *n* (%)	226 (69.3)	161 (66.8)	65 (76.5)	0.127
Diabetes, *n* (%)	101 (31.0)	73 (30.3)	28 (32.3)	0.750
Dyslipidemia, *n* (%)	71 (21.8)	52 (21.6)	19 (22.4)	1.000
Smoking ever, *n* (%)	80 (24.5)	57 (12.7)	23 (27.1)	0.630
Prior myocardial infarction, *n* (%)	30 (9.2)	15 (6.2)	15 (17.6)	0.004
Heart failure, *n* (%)	55 (16.9)	40 (16.6)	15 (17.6)	0.957
Chronic lung disease, *n* (%)	63 (19.3)	45 (18.7)	18 (21.2)	0.732
Impaired kidney function, *n* (%)	45 (13.8)	27 (11.2)	18 (21.2)	0.035
Immunodeficiency, *n* (%)	69 (21.2)	44 (18.3)	25 (29.4)	0.044
SpO2 (%)	95 (92–97)	95 (93–97)	92 (87–96)	<0.001

Continuous variables are expressed as mean ± standard deviation (SD) or median with interquartile range (IQR), as deemed appropriate. Categorical variables are expressed as absolute numbers and percentages. Abbreviations: BMI = body mass index, SpO2 = oxygen saturation.

**Table 2:** Laboratory characteristics of the patients.

	All Patients (*n* = 326)	No Clinical Deterioration (*n* = 241)	Clinical Deterioration (*n* = 85)	*p* Value
Lymphopaenia, *n* (%) (*n* = 240, 85)	145 (44.6)	100 (41.7)	45 (52.9)	0.095
White blood cell count (G/L) (*n* = 241,84)	6.76 (4.91–9.30)	6.27 (4.68–8.48)	7.97 (5.89–11.37)	<0.001
Elevated liver enzymes, *n* (%) (*n* = 230, 80)	193 (59.2)	137 (59.6)	56 (70.0)	0.127
LDH (U/L) (*n* = 219, 74)	275.0 (204.0–398.0)	241.0 (192.5–339.5)	448.5 (286.0–627.5)	<0.001
CRP (mg/L) (*n* = 241, 85)	82.5 (28.5–139.4)	62.8 (20.1–107.9)	140.4 (87.6–226.7)	<0.001
Ferritin (ng/L) (*n* = 213, 72)	557.0 (304.0–1004.0)	683.6 (298.0–859.0)	835.5 (406.8–1308.2)	<0.001
D-dimer (μg/mL) (*n* = 192, 72)	1.17 (0.62–2.62)	0.92 (0.58–1.68)	2.50 (1.41–4.24)	<0.001
Prothrombin time (sec) (*n* = 196, 67)	9.0 (8.5–9.6)	9.0 (8.5–9.5)	9.2 (8.6–9.9)	0.140
High sensitivity troponin T (ng/L) (*n* = 194, 71)	15.0 (7.0–36.0)	12.0 (6.0–27.0)	36.0 (19.0–74.0)	0.144
Creatine-kinase (U/L) (*n* = 176, 60)	80.0 (39.8–201.2)	72.0 (40.8–162.8)	113.0 (35.0–378.8)	0.071

Not all patients had blood laboratory results available. The *n* values indicate the number of patients who had blood samples collected for these laboratory metrics. The first *n* value is for those who had no clinical deterioration, the second *n* is for those with clinical deterioration. Categorical variables are expressed as absolute numbers and percentages. Continuous variables are expressed as median with interquartile range (IQR). Lymphopaenia is defined as lymphocyte count under 1 Giga/L. Abbreviations: CRP = C-reactive protein, LDH = lactate dehydrogenase.

**Table 3:** AI-based chest CT quantitative measurements.

	All Patients (*n* = 326)	No Clinical Deterioration (*n* = 241)	Clinical Deterioration (*n* = 85)	*p* Value
Lobe volume (mL)				
Right upper lobe	773.0 (585.8–925.2)	788.5 (628.5–942.0)	688.0 (541.5–908.5)	0.017
Right middle lobe	374.0 (287.0–492.0)	387.0 (292.0–500.0)	342.0 (265.2–481.2)	0.064
Right lower lobe	792.0 (611.8–1023.0)	806.0 (629.0–1044.0)	748.0 (578.0–981.5)	0.177
Left upper lobe	966.0 (774.0–1215.0)	990.0 (796.2–1231.2)	895.0 (725.0–1177.0)	0.029
Left lower lobe	763–0 (565.5–991.0)	786.0 (589.0–2138.0)	690.5 (518.2–861.5)	0.016
Affected area (%)				
Total	6.8 (1.9–22.1)	5.6 (1.5–16.6)	21.0 (6.2–45.0)	<0.001
Right upper lobe	2.7 (0.2–17.3)	1.2 (0.1–10.0)	13.2 (1.5–45.7)	<0.001
Right middle lobe	1.9 (0.1–12.2)	1.3 (0.0–7.9)	12.3 (0.9–37.4)	<0.001
Right lower lobe	12.9 (2.8–40.6	9.9 (1.9–27.4)	40.1 (12.3–63.1)	<0.001
Left upper lobe	2.0 (0.1–15.8)	1.4 (0.1–9.1)	8.9 (1.1–34.8)	<0.001
Left lower lobe	9.4 (1.5–37.3)	5.9 (0.9–26.8)	28.9 (4.8–60.6)	<0.001

Values are expressed as median with interquartile ranges.

**Table 4:** Severity scores calculated by the deep learning model based on the quantitative measurements.

	All Patients (*n* = 326)	No Clinical Deterioration (*n* = 241)	Clinical Deterioration (*n* = 85)	*p* Value
Severity score				
Total	7.0 (4.0–12.0)	6.0 (3.0–10.0)	11.0 (7.0–17.3)	<0.001
Right upper lobe	1.0 (0.0–2.0)	1.0 (0.0–2.0)	2.0 (1.0–3.3)	<0.001
Right middle lobe	1.0 (0.0–2.0)	1.0 (0.0–2.0)	2.0 (1.0–3.0)	<0.001
Right lower lobe	2.0 (1.0–3.0)	2.0 (1.0–3.0)	3.0 (1.0–4.0)	<0.001
Left upper lobe	1.0 (0.0–2.0)	1.0 (0.0–2.0)	2.0 (1.0–3.0)	<0.001

Values are expressed as median with interquartile ranges.

**Table 5:** Association between clinical and AI-based CT parameters with clinical deterioration.

	Unadjusted Analysis			Model 1: Clinical Parameters			Model 2: Clinical + AI-Based CT Parameters		
	OR	95% CI	*p*	OR	95% CI	*p*	OR	95% CI	*p*
Age	1.02	1.00–1.04	0.022	1.00	0.98–1.03	0.765	1.01	0.99–1.03	0.477
Male	0.98	0.60–1.61	0.935						
BMI	0.94	0.87–1.02	0.146						
Hypertension	1.61	0.93–2.91	0.098						
Diabetes	1.13	0.66–1.91	0.650						
Dyslipidemia	1.05	0.57–1.87	0.882						
Smoking ever	1.20	0.67–2.09	0.531						
Prior myocardial infarction	3.23	1.50–7.00	0.003	3.31	1.37–8.12	0.008	2.81	1.12–7.04	0.027
Heart failure	1.08	0.55–2.03	0.824						
Chronic lung disease	1.17	0.62–2.13	0.615						
Impaired kidney function	2.13	1.09–4.08	0.024	2.03	0.93–4.41	0.074	2.15	0.96–4.78	0.059
Immunodeficiency	1.87	1.05–3.28	0.032	1.66	0.84–3.23	0.138	2.08	1.02–4.25	0.043
SpO2	0.90	0.86–0.94	<0.001	0.94	0.89–0.98	0.005	0.96	0.91–1.00	0.060
CRP *	2.25	1.76–2.96	<0.001	1.95	1.51–2.58	<0.001	1.73	1.32–2.33	<0.001
CT severity score	1.15	1.10–1.20	<0.001				1.08	1.02–1.15	0.013

Model 1: clinical parameters: age, prior myocardial infarction, impaired kidney function, immunodeficiency, and SpO2 at the time hospital admission. Model 2: Model 1 + AI-based CT severity score. * Odds ratios are per two-fold increase of the variable. Abbreviations: AI = artificial intelligence; BMI = body mass index; CI = confidence interval; CT = computed tomography; OR = odds ratio.

## Data Availability

The data presented in this study are available on request from the corresponding author.

## References

[1] Kang S.J., Jung S.I. (2020). Age-Related Morbidity and Mortality among Patients with COVID-19. Infect. Chemother..

[2] Dong E., Du H., Gardner L. (2020). An interactive web-based dashboard to track COVID-19 in real time. Lancet Infect. Dis..

[3] Merkely B., Szabo A.J., Kosztin A., Berenyi E., Sebestyen A., Lengyel C., Merkely G., Karady J., Varkonyi I., Papp C. (2020). Novel coronavirus epidemic in the Hungarian population, a cross-sectional nationwide survey to support the exit policy in Hungary. Geroscience.

[4] Abbasi B., Akhavan R., Ghamari Khameneh A., Zandi B., Farrokh D., Pezeshki Rad M., Feyzi Laein A., Darvish A., Bijan B. (2021). Evaluation of the relationship between inpatient COVID-19 mortality and chest CT severity score. Am. J. Emerg. Med..

[5] Ozel M., Aslan A., Arac S. (2021). Use of the COVID-19 Reporting and Data System (CO-RADS) classification and chest computed tomography involvement score (CT-IS) in COVID-19 pneumonia. Radiol. Med..

[6] Lessmann N., Sanchez C.I., Beenen L., Boulogne L.H., Brink M., Calli E., Charbonnier J.P., Dofferhoff T., van Everdingen W.M., Gerke P.K. (2021). Automated Assessment of COVID-19 Reporting and Data System and Chest CT Severity Scores in Patients Suspected of Having COVID-19 Using Artificial Intelligence. Radiology.

[7] Luo X., Zhou W., Yan X., Guo T., Wang B., Xia H., Ye L., Xiong J., Jiang Z., Liu Y. (2020). Prognostic Value of C-Reactive Protein in Patients With Coronavirus 2019. Clin. Infect. Dis..

[8] Wang G., Wu C., Zhang Q., Wu F., Yu B., Lv J., Li Y., Li T., Zhang S., Wu C. (2020). C-Reactive Protein Level May Predict the Risk of COVID-19 Aggravation. Open Forum Infect. Dis..

[9] Wang L. (2020). C-reactive protein levels in the early stage of COVID-19. Med. Mal. Infect..

[10] Wu C., Chen X., Cai Y., Xia J., Zhou X., Xu S., Huang H., Zhang L., Zhou X., Du C. (2020). Risk Factors Associated With Acute Respiratory Distress Syndrome and Death in Patients With Coronavirus Disease 2019 Pneumonia in Wuhan, China. JAMA Intern. Med..

[11] Knight S.R., Ho A., Pius R., Buchan I., Carson G., Drake T.M., Dunning J., Fairfield C.J., Gamble C., Green C.A. (2020). Risk stratification of patients admitted to hospital with covid-19 using the ISARIC WHO Clinical Characterisation Protocol: Development and validation of the 4C Mortality Score. BMJ.

[12] Xie J., Covassin N., Fan Z., Singh P., Gao W., Li G., Kara T., Somers V.K. (2020). Association Between Hypoxemia and Mortality in Patients With COVID-19. Mayo Clin. Proc..

[13] Wang X., Fang X., Cai Z., Wu X., Gao X., Min J., Wang F. (2020). Comorbid Chronic Diseases and Acute Organ Injuries Are Strongly Correlated with Disease Severity and Mortality among COVID-19 Patients: A Systemic Review and Meta-Analysis. Research.

[14] Hendren N.S., de Lemos J.A., Ayers C., Das S.R., Rao A., Carter S., Rosenblatt A., Walchok J., Omar W., Khera R. (2021). Association of Body Mass Index and Age With Morbidity and Mortality in Patients Hospitalized With COVID-19: Results From the American Heart Association COVID-19 Cardiovascular Disease Registry. Circulation.

[15] Lee S.C., Son K.J., Han C.H., Jung J.Y., Park S.C. (2020). Impact of comorbid asthma on severity of coronavirus disease (COVID-19). Sci. Rep..

[16] Akbariqomi M., Hosseini M.S., Rashidiani J., Sedighian H., Biganeh H., Heidari R., Moghaddam M.M., Farnoosh G., Kooshki H. (2020). Clinical characteristics and outcome of hospitalized COVID-19 patients with diabetes: A single-center, retrospective study in Iran. Diabetes Res. Clin. Pract..

[17] Ciceri F., Castagna A., Rovere-Querini P., De Cobelli F., Ruggeri A., Galli L., Conte C., De Lorenzo R., Poli A., Ambrosio A. (2020). Early predictors of clinical outcomes of COVID-19 outbreak in Milan, Italy. Clin. Immunol..

[18] Gupta S., Hayek S.S., Wang W., Chan L., Mathews K.S., Melamed M.L., Brenner S.K., Leonberg-Yoo A., Schenck E.J., Radbel J. (2020). Factors Associated With Death in Critically Ill Patients With Coronavirus Disease 2019 in the US. JAMA Intern. Med..

[19] Jackson B.R., Gold J.A.W., Natarajan P., Rossow J., Neblett Fanfair R., da Silva J., Wong K.K., Browning S.D., Bamrah Morris S., Rogers-Brown J. (2020). Predictors at admission of mechanical ventilation and death in an observational cohort of adults hospitalized with COVID-19. Clin. Infect. Dis..

[20] Shi S., Qin M., Cai Y., Liu T., Shen B., Yang F., Cao S., Liu X., Xiang Y., Zhao Q. (2020). Characteristics and clinical significance of myocardial injury in patients with severe coronavirus disease 2019. Eur. Heart J..

[21] Li Q., Chen L., Li Q., He W., Yu J., Chen L., Cao Y., Chen W., Di W., Dong F. (2020). Cancer increases risk of in-hospital death from COVID-19 in persons <65 years and those not in complete remission. Leukemia.

[22] Dai M., Liu D., Liu M., Zhou F., Li G., Chen Z., Zhang Z., You H., Wu M., Zheng Q. (2020). Patients with Cancer Appear More Vulnerable to SARS-CoV-2: A Multicenter Study during the COVID-19 Outbreak. Cancer Discov..

[23] Madjid M., Vela D., Khalili-Tabrizi H., Casscells S.W., Litovsky S. (2007). Systemic infections cause exaggerated local inflammation in atherosclerotic coronary arteries: Clues to the triggering effect of acute infections on acute coronary syndromes. Tex. Heart Inst. J..

[24] Jee J., Stonestrom A.J., Devlin S., Nguyentran T., Wills B., Narendra V., Foote M.B., Lumish M., Vardhana S.A., Pastores S.M. (2021). Oncologic immunomodulatory agents in patients with cancer and COVID-19. Sci. Rep..

[25] Jee J., Foote M.B., Lumish M., Stonestrom A.J., Wills B., Narendra V., Avutu V., Murciano-Goroff Y.R., Chan J.E., Derkach A. (2020). Chemotherapy and COVID-19 Outcomes in Patients With Cancer. J. Clin. Oncol..

[26] Mortensen R.F. (2001). C-reactive protein, inflammation, and innate immunity. Immunol. Res..

[27] Coster D., Wasserman A., Fisher E., Rogowski O., Zeltser D., Shapira I., Bernstein D., Meilik A., Raykhshtat E., Halpern P. (2020). Using the kinetics of C-reactive protein response to improve the differential diagnosis between acute bacterial and viral infections. Infection.

[28] Zhu N., Zhang D., Wang W., Li X., Yang B., Song J., Zhao X., Huang B., Shi W., Lu R. (2020). A Novel Coronavirus from Patients with Pneumonia in China, 2019. N. Engl. J. Med..

[29] Chassagnon G., Vakalopoulou M., Battistella E., Christodoulidis S., Hoang-Thi T.N., Dangeard S., Deutsch E., Andre F., Guillo E., Halm N. (2021). AI-driven quantification, staging and outcome prediction of COVID-19 pneumonia. Med. Image Anal..

[30] Murphy K., Smits H., Knoops A.J.G., Korst M., Samson T., Scholten E.T., Schalekamp S., Schaefer-Prokop C.M., Philipsen R., Meijers A. (2020). COVID-19 on Chest Radiographs: A Multireader Evaluation of an Artificial Intelligence System. Radiology.

[31] Li L., Qin L., Xu Z., Yin Y., Wang X., Kong B., Bai J., Lu Y., Fang Z., Song Q. (2020). Using Artificial Intelligence to Detect COVID-19 and Community-acquired Pneumonia Based on Pulmonary CT: Evaluation of the Diagnostic Accuracy. Radiology.

